# High fish intake rich in n-3 polyunsaturated fatty acids reduces cardiovascular disease incidence in healthy adults: The ATTICA cohort study (2002-2022)

**DOI:** 10.3389/fphys.2023.1158140

**Published:** 2023-03-28

**Authors:** Elena Critselis, Thomas Tsiampalis, Evangelia Damigou, Ekavi Georgousopoulou, Fotios Barkas, Christina Chrysohoou, John Skoumas, Christos Pitsavos, Evangelos Liberopoulos, Costas Tsioufis, Petros P. Sfikakis, Demosthenes Panagiotakos

**Affiliations:** ^1^ Department of Nutrition and Dietetics, School of Health Sciences and Education, Harokopio University, Athens, Greece; ^2^ Department of Primary Care and Population Health, University of Nicosia Medical School, Nicosia, Cyprus; ^3^ Discipline of Nutrition & Dietetics, Faculty of Health, University of Canberra, Canberra, ACT, Australia; ^4^ First Cardiology Clinic, School of Medicine, National and Kapodistrian University of Athens, Athens, Greece; ^5^ First Department of Propaedeutic Internal Medicine, School of Medicine, Laiko General Hospital, National and Kapodistrian University of Athens, Athens, Greece

**Keywords:** mediterranean diet, nutrition, fish, fish oils, omega-3 fatty acids, cardiovascular disease, incidence, mortality

## Abstract

**Background:** The long-term effects of high fish intake rich in n-3 fatty acids for deterring cardiovascular disease (CVD) and related adverse outcomes in healthy individuals have not been yet elucidated.

**Purpose:** To evaluate the association between total seafood, as well as small fish, intake on 10- and 20-year CVD incidence and mortality in healthy adults.

**Methods:** A prospective cohort study (n = 2,020) was conducted in healthy community dwelling adults in Athens, Greece, selected following age- and sex-based random multistage sampling (mean ± SD age at baseline: 45.2 ± 14.0 years). Seafood (high (>2 servings/week) vs. low (≤2 servings/week) intake), including small fish rich in n-3 fatty acids (high (>1 serving/week) vs. low (≤1 serving/week) intake), consumption was evaluated by semi-quantitative food frequency questionnaire at baseline. The occurrence of non-fatal and/or fatal CVD events (ICD-10) was assessed during 10- and 20-year follow-up periods.

**Results:** Only 32.7% and 9.6% of participants had high seafood and small fish intakes, respectively. Participants with high seafood intake had 27% decreased 10-year CVD risk (adj. HR:0.73; 95% CI:0.55-0.98) and 74% lower attributable mortality (adj. HR:0.26; 95% CI:0.11-0.58). Participants with high seafood intake also sustained a 24% lower 20-year risk of CVD mortality (adj. HR: 0.76; 95% CI: 0.55-0.98). Moreover, participants with high small fish intake had a lower 10-year CVD risk and 76% decreased risk of 10-year CVD mortality (adj. HR:0.24; 95% CI:0.06-0.99), even among normotensive individuals (adj. HR:0.31; 95% CI:0.13-0.73). When analogous analyses focused on 20-year CVD incidence and mortality, similar but not significant associations were observed (all *p*-values >0.10).

**Conclusion:** High intake of seafood, and particularly small fish rich in n-3 fatty acids, was associated with a lower risk of 10-year fatal and non-fatal CVD. Thus, public health interventions aimed at enhancing small fish consumption may most effectively deter long-term CVD outcomes, particularly among low risk normotensive individuals.

## 1 Introduction

Cardiovascular diseases (CVD) have emerged as a prevailing cause of morbidity and mortality worldwide (Ritchey et al., 2014; Wall et al., 2018). Frequent consumption of fish, shellfish, and molluscs constitutes a vital component of the Mediterranean Diet and is essential for ensuring cardiometabolic health (Mozaffarian and Rimm, 2006). The Seven Countries Study initially established that frequent seafood consumption deters CVD onset and further progression to coronary heart disease (CHD) and attributable mortality (Menotti et al., 1999). Subsequent large prospective cohort studies revealed that even modest (1 serving/week), as well as high (2–4 servings/week), fish consumption levels were associated with a 15% reduction in CVD risk (Virtanen et al., 2008), as well as diminishment in adverse related health outcomes (Jayedi and Shab-Bidar, 2020). In fact, recent umbrella reviews of meta-analyses conducted among prospective cohort studies suggest that a dose-response relationship exists since every 100 g/d increment in fish consumption substantially reduces the risk of a wide array of CVD events, including CHD (summary relative risk, SRR: 0.88; 95% CI: 0.79–0.99), heart failure (SRR: 0.80; 95% CI: 0.67–0.95), myocardial infarction (SRR: 0.75; 95% CI: 0.65–0.93), and CVD mortality (SRR: 0.75; 95% CI: 0.65–0.87) (Jayedi and Shab-Bidar, 2020). Due to its apparent cardioprotective effects, even modest fish consumption is deemed as a healthy animal-based dietary source of protein (Virtanen et al., 2008; Jayedi and Shab-Bidar, 2020) for securing optimal health outcomes and reducing all-cause mortality (English et al., 2021). However, most recent highest quality systematic reviews and meta-analyses suggest that the impact of fish consumption is in fact limited for reducing CVD mortality (RR: 0.96; 95% CI: 0.94–0.98) (Kwok et al., 2019), and hence remains controversial.

In particular, recent meta-analytic findings question whether overall seafood consumption *per se*, or particular fish, render beneficial cardiometabolic effects. Specifically, meta-analytic findings show that while fatty fish consumption is inversely associated with CHD incidence (RR: 0.92; 95% CI: 0.86–0.97) and attributable mortality (RR: 0.83; 95% CI: 0.70–0.98), such associations were not sustained when lean fish consumption was examined (Giosue et al., 2022). It is posited that the beneficial health effects of fish consumption are primarily attributed to elevated levels of polyunsaturated fatty acids (PUFAs), including, namely, eicosapentaenoic (EPA) (20:5 n–3) and docosahexaenoic (DHA) (22:6 n–3) acids (also known as long chain n-3 or omega-3 PUFAs), which are abundantly found in fatty fish, such as small fish (including anchovy, sardine and mackerel) readily found and consumed in the Mediterranean region, with typical serving sizes and/or meals rendering approximately 1.5–2 g of n-3 fatty acids essential for CVD prevention (Mozaffarian and Rimm, 2006).

Hence, to date, discordant related dietary guidelines targeting CVD prevention exist, varying with regard to overall and/or specific fish intake recommendations. For example, the American Heart Association (AHA) encourages the consumption of 1–2 seafood based meals per week to reduce CVD risk and death (Rimm et al., 2018). In contrast, the Sixth Joint Task Force of the European Society of Cardiology and Other Societies on Cardiovascular Disease Prevention in Clinical Practice recommend that beyond consuming seafood 1 to 2 times per week, one such meal ought to include fatty fish (Piepoli et al., 2016). Moreover, particularly given contradictory observational study findings and risk assessments regarding potential contaminants present in large fatty fish (Ferrante et al., 2019; Danopoulos et al., 2020), the utility of recommending high intakes of n-3 fatty acid rich fish (as opposed to total seafood) for CVD prevention in healthy individuals remains questionable (Giosue et al., 2022). As a result, such recommendations are often endorsed solely in specific high CVD risk patient groups (Mohan et al., 2021). The long-term effects of high fish intake rich in n-3 fatty acids for deterring CVD and related adverse outcomes in healthy individuals have not been yet elucidated (Siscovick et al., 2017). Such evidence could inform the development of optimal dietary guidelines tailored for cardiovascular health (Kwok et al., 2019) in the general population.

The present study aimed to evaluate the association between total seafood, as well as small fatty fish rich in n-3 fatty acids, on 10- and 20-year CVD incidence and mortality in the ATTICA Cohort Study.

## 2 Materials and methods

### 2.1 Study sample

As previously detailed (Panagiotakos et al., 2015), the ATTICA Cohort Study is a prospective, observational cohort investigation which assesses cardiovascular disease outcomes over a 10- and 20-year period in community dwelling healthy individuals. At baseline (2001-2002), random multistage sampling, based on the age and sex distribution of the population according to the 2001 National Census Survey, was conducted in residents of the greater metropolitan area of Athens, Greece (including 78% urban municipalities). One participant per household was enrolled, whilst institutionalized individuals were excluded. Of 4,056 initially invited individuals, 3,042 healthy community dwelling volunteers agreed to participate (75% participation rate), of which 1,514 males (mean age ±standard deviation (SD): 46 ± 13 years; range 18–87 years) and 1,528 females (mean age ±SD: 45 ± 13 years; range: 18–89 years) (Pitsavos et al., 2003). Following a detailed clinical evaluation by trained physicians, individuals with pre-existing CVD were excluded from further participation.

### 2.2 Baseline assessment

#### 2.2.1 Demographic and lifestyle characteristics

Face-to-face interviews were conducted according to standardized questionnaires regarding demographic characteristics (e.g., age, sex, highest attained educational level, and income), detailed medical history, and lifestyle habits (e.g., dietary habits, smoking history and habitual/leisure time physical activity).

For highest attained educational level, participants were categorized into the following categories: a) low (i.e., <12 years education), b) moderate (i.e., including 13–16 years education); and c) high (i.e., >16 years education) educational level. “Current smokers” smoked ≥1 cigarette per day, “never smokers” had never smoked, and “former smokers” had ceased smoking ≥1 year prior to baseline.

The International Physical Activity Questionnaire (IPAQ) (Papathanasiou et al., 2009) was used as an index of weekly energy expenditure from physical activity; being physically active was defined as >3 MET/day of leisure-time activity, of specific intensity and duration, during the past year. Otherwise, subjects were identified as physically inactive.

#### 2.2.2 Dietary assessment

Dietary habits were assessed based on a validated semi-quantitative food-frequency questionnaire (FFQ) (Katsouyanni et al., 1997) wherein participants reported the average weekly or daily intakes of 156 food items and beverages consumed during the past year. Frequency of consumption of foods (including fish and seafood) and beverages was categorized as: a) “never or less than once per month”; b) “1-3 times per month”; c) “1 time per week”; d) “2 times per week,” “3-4 times per week”; e) “5-6 times per week”; f) “1 time per day”; and g) “≥2 times per day.” Approximate monthly frequency of food item consumption was calculated. Block photographs were employed for assisting responders to define portion sizes of foods and beverages. Frequency of foods or beverages consumed were subsequently expressed as: a) “0 servings per week”; b) “0.5 servings per week”; c) “1 serving per week”; d) “2 servings per week”; e) “3.5 servings per week”; f) “5.5 servings per week”; g) “7 servings per week”; and h) “14 servings per week.” Composite scores were employed to describe overall dietary patterns. The Mediterranean Diet Score (MedDietScore, score range 0–55) was used to depict adherence to dietary patterns proximal to those of the Mediterranean Diet as follows: a) low adherence: <27 units, and b) high adherence: ≥27 units (Willett et al., 1995; Panagiotakos et al., 2006).

For the present analysis, we considered the following categories of fish, seafood and shellfish intake based on readily available foods and food products in Greece: a) total seafood intake, including marine fish, shellfish and molluscs; and, b) total intake of small fish rich in n-3 fatty acids. Total seafood intake was computed as the total intake of lean fish and fish products (e.g., white fish such as cod, haddock, and plaice, with fat content <4 g/100 g), large fatty fish and fish products (e.g., including salmon, tuna, and trout, with fat content ranging 4–14 g/100 g), small fatty fish (see below), and shellfish or molluscs and their related products (e.g., crab, mussels, octopus, and shrimp). In addition, total small fish rich in n-3 fatty acids included related fish and fish products abundant in the Mediterranean region, including anchovy, sardine, and mackerel (Fatty Acids, 1998; Welch et al., 2002). Based on FFQ responses, participants were classified into the following weekly seafood consumption categories defined according to the Greek National Dietary Guidelines (corresponding to 150 g fish or seafood per serving) (Kastorini et al., 2019): (i) low intake (≤2 servings/week), or (ii) high intake (>2 servings/week). Finally, regarding the consumption of small fish rich in n-3 fatty acids, subjects were categorized as consuming either 1) low intake (≤1 serving/week), or 2) high intake (>1 serving/week), according to Greek National Dietary Guidelines recommendations (Kastorini et al., 2019).

#### 2.2.3 Anthropometric and clinical assessments

As detailed elsewhere (Panagiotakos et al., 2015), weight, height, waist, and hip circumferences were measured according to standard procedures by trained personnel. Weight (kg) and standing height (m^2^) were used to calculate body mass index (BMI). Subjects with BMI >29.9 kg/m^2^ were defined as obese. Waist and hip circumferences (cm) were used to calculate waist-to-hip ratio.

While in a sitting position, subjects’ arterial blood pressure was measured blindly three times by a trained cardiologist while the participant’s right arm was relaxed and well supported by a table, at 45^o^ from the trunk (ELKA aneroid manometric sphygmometer, Von Schlieben Co, West Germany). Systolic (SBP) and diastolic (DBP) blood pressure levels were determined by the first perception of sound (of tapping quality) and phase V (fully muffed repetitive sounds). An average of the three measurements was recorded. Individuals with SBP ≥140 mm Hg, DBP >90 mm Hg, and/or taking antihypertensive medication, were identified as hypertensive.

#### 2.2.4 Laboratory assessments

Morning blood samples were collected following a 12 h fast from participants’ antecubital vein. Blood lipids (i.e., serum total cholesterol) were measured using chromatographic enzymic method in a RA-1000 Technicon automatic analyzer (*Dade Behring, Marburg, Germany*). Hypercholesterolemia was defined as >220 mg/dL total cholesterol levels or hypolipidemic medication use. Blood glucose levels (mg/dL) were measured with a Beckman Glucose Analyzer (Beckman Instruments, Fullerton, CA, United States). Diabetes mellitus was defined as fasting blood sugar levels >125 mg/dL or antidiabetic medication use. Metabolic syndrome was defined by the National Cholesterol Education Program Adult Treatment panel III (revised NCEP ATP III) definition (Lipsy, 2003).

### 2.3 10- and 20-year follow-up assessment

Follow-up evaluation was conducted in two waves, i.e., at 10- and 20-years following baseline assessment. Of 3,042 initially enrolled participants, 10-year follow-up evaluation was achieved in 2,583 participants (85% participation rate, with mean baseline age (mean years ±SD): 45 ± 14 years and 46 ± 14 years for women and men, respectively, with no difference as compared to the overall study sample), and 20-year follow-up was achieved in 2,169 participants (71% participation rate), with mean baseline age (mean years ±SD): 45 ± 14 years and 43 ± 13 years for women and men, respectively. Of participants lost to follow-up, about 10% could not be traced due to missing or erroneous contact information and another 10% denied to participate. No significant differences were observed regarding age and sex distribution, baseline smoking habits, physical activity levels, and dietary habits between those who participated and those who were lost to follow-up (all *p* values > 0.50). Complete CVD evaluation was achieved in 2,020 participants at 10-years and 1,988 at 20-years follow-up. Follow-up evaluation included retrieving participants’ detailed medical records. If lacking, face-to-face interviews were conducted by trained study investigators. The follow-up evaluation regarded: (a) vital status (death from any cause or due to CVD), or (b) development of coronary heart disease (including myocardial infarction, angina pectoris, other identified forms of ischemia -WHO-ICD coding 410–414.9, 427.2, 427.6-, heart failure of different types, and chronic arrhythmias -WHO-ICD coding 400.0-404.9, 427.0–427.5, 427.9-) or development of stroke (WHO-ICD coding 430-438).

### 2.4 Statistical analysis

Categorical variables are presented as relative frequencies (%) and continuous variables are presented as mean values (±SD). Normality of the continuous characteristics’ distribution was tested through P-P plots and the Shapiro-Wilks test. Associations between the categorical characteristics and the weekly consumption of total fish and seafood, as well as small fish rich in omega 3 fatty acids, were evaluated through the Pearson Chi-squared test, while the independent samples t-test was used for comparing continuous normally distributed variables. Univariable Cox proportional-hazards regression was used to estimate hazard ratios (HRs) and 95% confidence intervals (95% CI) evaluating the association of total weekly intake of fish and seafood, as well as only total weekly intake of small fish rich in omega 3 fatty acids, with the 10- and 20-year occurrence of CVD and CVD attributable mortality. Multivariable regression analyses of the aforementioned models entailed adjusting for participants’ demographic, lifestyle, anthropometric and clinical characteristics, including age (years), sex, highest educational level attained, smoking habits (current smoker vs. non-smoker or former smoker), physical activity (physically active vs. inactive), BMI (kg/m^2^), family history of CVD (yes vs. no), as well as personal history of diabetes mellitus (yes vs. no), hypercholesterolemia (yes vs. no), and/or hypertension (yes vs. no). Time to each CVD event and death due to any CVD was recorded on an annual basis. Correlation Matrix of the models’ estimates was used to assess multicollinearity between independent variables. The proportional hazards assumption was tested by including interaction terms between fish consumption (both the total consumption, as well as the consumption of small fish), and the calendar year. Finally, subgroup analysis was also conducted in terms of the participants’ age (<65 years vs. ≥ 65 years), sex, adherence to the Mediterranean Diet (low vs. high adherence), and presence of hypertension (hypertensive vs. non-hypertensive). All statistical analyses were performed using STATA software (version 17.0, TStat S.r.I., Italy) and the criterion of statistical significance was a two-tailed *p*-value<0.05.

## 3 Results

### 3.1 Baseline characteristics

As depicted in Table 1, approximately one-third (32.7%) of participants adhered to the Greek National Dietary Guidelines recommendations and consumed >2 servings of marine fish and seafood on a weekly basis, while less than one-10th (9.6%) consumed >1 serving of small fish rich in n-3 fatty acids per week. Participants with high total seafood intake (>2 servings per week), as compared to their low intake counterparts (≤2 servings per week), were significantly more likely to be male (54.7% vs. 47.4%; *p* = 0.002), of younger age (*p* < 0.001), and of higher educational and SES status (*p* < 0.001). They also more frequently adhered to a healthy dietary pattern such as the Mediterranean Diet (*p* < 0.001) and reported higher levels of physical activity level (*p* = 0.031). Moreover, they presented less frequently with hypercholesterolemia and diabetes (*p*-values<0.001). While participants consuming high intakes of small fatty fish were also significantly more often male (58% vs. 48.9%; *p* = 0.016) and of younger age (*p* = 0.010), they were not found to differ from their low intake counterparts with respect to any other anthropometric and/or clinical parameters at baseline (Table 1 *).*

**TABLE 1:** Baseline demographic, lifestyle, anthropometric and clinical characteristics of the study population according to total weekly consumption of fish and seafood: The ATTICA Cohort Study, 2002-2012.

	Total sample	Total weekly consumption of marine fish and seafood			Total weekly consumption of small fish rich in omega 3 fatty acids		
	(N = 2,020)	≤2 servings/week (N = 1,360)	>2 servings/week (N = 660)	*p*-value	≤1 serving/week (N = 1,827)	>1 serving/week (N = 193)	*p*-value
**Demographic characteristicss**							
Age [in years; Mean (SD)]	45.2 (14.0)	47.6 (14.5)	40.1 (11.2)	**<0.001**	45.4 (14.2)	43.2 (10.6)	**0.010**
Female sex (n, %)	1,014 (50.2)	715 (52.6)	299 (45.3)	**0.002**	934 (51.1)	80 (41.4)	**0.016**
Low level of education (n, %)	375 (18.6)	316 (23.2)	59 (8.9)	**<0.001**	345 (18.9)	30 (15.5)	0.167
Low socioeconomic status (n, %)	344 (17.0)	294 (21.6)	50 (7.5)	**<0.001**	278 (15.2)	27 (13.8)	0.699
**Lifestyle characteristics**							
Current smoker (n, %)	826 (40.9)	533 (39.2)	293 (44.4)	**0.026**	753 (41.2)	73 (37.8)	0.447
Level of adherence to the Mediterranean diet- MedDietScore [Mean (SD)]	25.9 (6.4)	25.4 (5.7)	26.8 (7.7)	**<0.001**	25.7 (6.2)	26.9 (8.5)	0.060
Physically active (n, %)	719 (35.6)	462 (34.0)	257 (38.9)	**0.031**	647 (35.4)	72 (37.3)	0.506
**Anthropometric characteristics**							
Body Mass Index [in kg/m^2^; Mean (SD)]	26.3 (4.5)	26.5 (4.5)	26.0 (4.6)	**0.020**	26.3 (4.5)	26.6 (4.4)	0.400
Obese (n, %)	378 (18.7)	268 (19.7)	110 (16.7)	0.105	338 (18.5)	40 (20.7)	0.446
Waist- Hip ratio [Mean (SD)]	0.9 (0.1)	0.9 (0.1)	0.9 (0.1)	0.630	0.9 (0.1)	0.9 (0.1)	0.963
**Clinical characteristics**							
Personal history of hypertension (n, %)	636 (31.5)	445 (32.7)	191 (28.9)	0.128	574 (31.4)	62 (32.1)	0.738
Personal history of hypercholesterolaemia (n, %)	861 (42.6)	657 (48.3)	204 (30.9)	**<0.001**	789 (43.2)	72 (37.3)	0.115
Personal history of diabetes mellitus (n, %)	145 (7.2)	120 (8.8)	25 (3.8)	**<0.001**	133 (7.3)	12 (6.2)	0.403
Family history of CVD (n, %)	574 (28.4)	393 (28.9)	181 (27.1)	0.701	519 (28.4)	55 (28.5)	0.961

Continuous characteristics are presented as mean (standard deviation (SD)) and categorical variables as relative frequencies; Level of education was categorized as: Low (0–6 years of education), Middle (7–12 years of education), High (≥13 years of education); Level of physical activity was measured in MET- minutes *via* the IPAQ questionnaire and participants with ≥150 metabolic equivalent - MET-minutes/week were classified as being physically active; Level of adherence to the Mediterranean diet was evaluated *via* the MedDietScore scale (range 0–55); Obesity was defined as Body Mass Index (BMI)≥ 30.0 kg/m^2^; Diabetes mellitus was defined as a fasting blood sugar >125 mg/dL or the use of antidiabetic medication; Patients whose average blood pressure levels were greater or equal to 140/90 mm Hg or were under antihypertensive medication were classified as hypertensives; The definition of hypercholesterolemia was based on the total serum cholesterol levels (≥200 mg/dL); *p*-values for the comparisons between the categories of weekly consumption of fish and seafood were derived from using the independent samples t-test (continuous variables) and the Pearson chi-squared test (categorical variables); *p*-values in bold represent the statistically significant differences (*p* < 0.05).

Significant associations are highlighted in bold.

### 3.2 10- and 20-year CVD incidence

**FIGURE 1:**
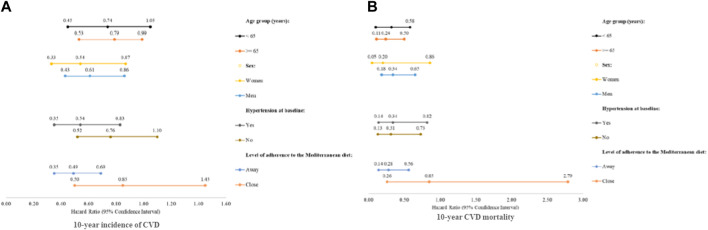
Hazard Ratio (HR) and 95% Confidence Interval (95% CI) for fatal and/or non-fatal 10 year CVD events according to weekly consumption of marine fish and seafood, stratified by participants’ age, sex, adherence to the Mediterranean diet, and history of hypertension: The ATTICA Cohort Study, 2002-2012. **(A)** Total weekly consumption of fish and seafood in relation to 10 year incidence of CVD. **(B)** Total weekly consumption of fish and seafood in relation to CVD mortality. The results are based on multivariable Cox proportional hazard model, adjusted for demographic characteristics [including age (years), sex, and highest educational level attained], lifestyle and anthropometric characteristics [including smoking habits (current smoker vs non-smoker / former smoker), physical activity (active vs sedentary), and BMI (kg/m^2^)], family history of CVD (yes vs no), and personal history of diabetes mellitus, hypercholesterolemia, and/or hypertension (yes vs no).

As depicted in Table 2, incidence of CVD events occurred less frequently among participants with a high, vs. low, intake of total marine fish and seafood (*p* = 0.001). Following adjustment for demographic, lifestyle and anthropometric characteristics, multivariable Cox regression analysis evaluating the association between total weekly seafood intake and subsequent CVD incidence revealed that participants who consumed >2 servings per week of seafood had at least 27% decreased risk of developing CVD at 10-years (adj. HR: 0.73; 95% CI: 0.55-0.98), and 18% decreased risk of developing CVD at 20-years of follow-up (adj. HR: 0.82; 95% CI: 0.66–1.00), albeit with the observed association being marginally sustained following further adjustment for personal and CVD family medical history (10-year adj. HR: 0.76; 95% CI: 0.56–1.02; 20-year adj. HR: 0.88; 95% CI: 0.73–1.06). Furthermore, as illustrated in Figure 1A, the beneficial role of high total seafood consumption upon 10-year CVD incidence was most prominent among women (adj. HR: 0.54; 95% CI: 0.35-0.87) and those aged ≥65 years old (adj. HR: 0.79; 95% CI: 0.53-0.99), as well as among those with low adherence to the Mediterranean Diet (adj. HR: 0.49; 95% CI: 0.35-0.69) or with hypertension at baseline (adj. HR: 0.54; 95% CI: 0.35-0.83). No significant associations were observed at 20-years of follow-up.

**TABLE 2:** Hazard Ratio (HR) and 95% Confidence Interval (95% CI) for fatal and/or non-fatal 10-year CVD events according to the weekly consumption of fish and seafood, as well as small fish rich in omega 3 fatty acids: The ATTICA Cohort Study, 2002-2012.

		Total weekly consumption of marine fish and seafood	
		≤2 servings/week	>2 servings/week
10- year CVD incidence	*Number of fatal or non-fatal CVD events/non-CVD events*	239/1121	77/583
	*Model 1* *: Univariable*	*Reference category*	0.62 (0.47, 0.81)**
	*Model 2* *: Adjusted for demographic characteristics*		0.72 (0.54, 0.95)**
	*Model 3* *: Model 2 + Adjusted for lifestyle and anthropometric characteristics*		0.73 (0.55, 0.98)**
	*Model 4* *: Model 3 + Adjusted for personal and family medical history*		0.76 (0.56, 1.02)*

		Total weekly consumption of small fish rich in omega 3 fatty acids	
		≤1 serving/week	>1 serving/week
	*Number of fatal or non-fatal CVD events/non-CVD events*	292/1535	25/168
	*Model 1* *: Univariable*	*Reference category*	0.78 (0.51, 1.21)
	*Model 2* *: Adjusted for demographic characteristics*		0.74 (0.48, 1.15)
	*Model 3* *: Model 2 + Adjusted for lifestyle and anthropometric characteristics*		0.73 (0.47, 1.13)
	*Model 4* *: Model 3 + Adjusted for personal and family medical history*		0.76 (0.48, 1.19)

		Total weekly consumption of marine fish and seafood	
		≤2 servings/week	>2 servings/week
10- year CVD mortality	*Number of fatal CVD events/non-CVD events*	65/1295	11/649
	*Model 1* *: Univariable*	*Reference category*	0.25 (0.12, 0.51)***
	*Model 2* *: Adjusted for demographic characteristics*		0.28 (0.14, 0.58)**
	*Model 3* *: Model 2 + Adjusted for lifestyle and anthropometric characteristics*		0.29 (0.14, 0.59)**
	*Model 4* *: Model 3 + Adjusted for personal and family medical history*		0.26 (0.11, 0.58)**

		Total weekly consumption of small fish rich in omega 3 fatty acids	
		≤1 serving/week	>1 serving/week
	*Number of fatal CVD events/non-CVD events*	75/1752	2/191
	*Model 1* *: Univariable*	*Reference category*	0.24 (0.06, 0.96)**
	*Model 2* *: Adjusted for demographic characteristics*		0.23 (0.06, 0.95)**
	*Model 3* *: Model 2 + Adjusted for lifestyle and anthropometric characteristics*		0.23 (0.06, 0.94)**
	*Model 4* *: Model 3 + Adjusted for personal and family medical history*		0.24 (0.06, 0.99)**

The results are based on multivariable Cox proportional hazard model, adjusted for demographic characteristics (including age (years), sex, and highest educational level attained), lifestyle and anthropometric characteristics (including smoking habits (current smoker vs. non-smoker/former smoker, physical activity (active vs. sedentary), and BMI (kg/m^2^)), family history of CVD (yes vs. no), and personal history of diabetes mellitus, hypercholesterolemia, and/or hypertension (yes vs. no). ***p< 0.001, **p< 0.05, *p< 0.10.

**FIGURE 2:**
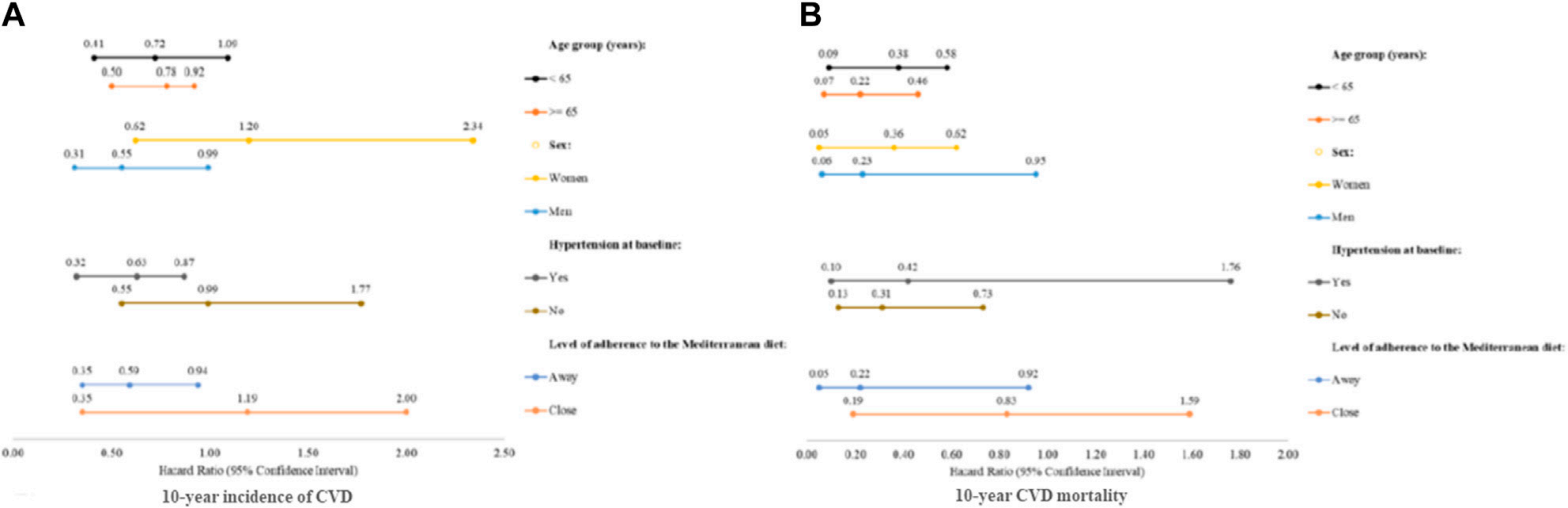
Hazard Ratio (HR) and 95% Confidence Interval (95% CI) for fatal and/or non-fatal 10 year CVD events according to the weekly consumption of small fish rich in omega 3 fatty acids, stratified by participants’ age, sex, adherence to the Mediterranean diet, and history of hypertension: The ATTICA Cohort Study, 2002-2012. **(A)** Total weekly consumption of small fish rich in omega 3 fatty acids in relation to 10 year incidence of CVD. **(B)** Total weekly consumption of small fish rich in fatty acids in relation to CVD mortality. The results are based on multivariable Cox proportional hazard model, adjusted for demographic characteristics (including age (years), sex, and highest educational level attained), lifestyle and anthropometric characteristics [including smoking habits (current smoker vs non-smoker / former smoker), physical activity (active vs sedentary), and BMI (kg/m^2^)], family history of CVD (yes vs no), and personal history of diabetes mellitus, hypercholesterolemia, and/or hypertension (yes vs no).

In contrast, participants with high consumption of small fish rich in n-3 fatty acids did not significantly differ from their respective counterparts with respect to either the 10- or 20-year CVD incidence (14.9% (>1 serving/week) vs. 19.0% (≤1 serving/week); *p* = 0.271). Furthermore, neither the univariable nor the multivariable regression models revealed a significant association between high intake of small fatty fish and subsequent occurrence of 10-year CVD. Even so, as illustrated in Figure 2A, the beneficial role of high small fatty fish consumption on 10-year CVD incidence was evident in men (adj. HR: 0.55; 95% CI: 0.31-0.99) and those aged ≥65 years old (adj. HR: 0.78; 95% CI: 0.50-0.92), as well as those with low adherence to the Mediterranean Diet (adj. HR: 0.59; 95% CI: 0.35-0.94) and/or with hypertension at baseline (adj. HR: 0.53; 95% CI: 0.32-0.87). No significant associations were observed at 20-years of follow-up.

### 3.3 10- and 20-year CVD mortality

As shown in Table 2, participants who consumed a high *vs* low, total seafood intake exhibited significantly less frequently 10-year CVD attributable mortality (1.7% vs. 5.0%, *p* < 0.001), while the 20-year CVD attributable mortality was 9.0% vs. 12.0%, respectively (*p* = 0.575). Moreover, after adjusting for several demographic, lifestyle and clinical characteristics, participants consuming >2 servings of marine fish and seafood on a weekly basis had a 74% lower 10-year risk of dying due to CVD (adj. HR: 0.26; 95% CI: 0.11-0.58), and a 24% lower 20-year risk of dying due to CVD (adj. HR: 0.76; 95% CI: 0.55-0.98). As illustrated in Figure 1B, the observed notable beneficial impact of consuming frequently (>2 servings per week) marine fish and seafood in deterring 10-year CVD attributable mortality was most apparent among women (adj. HR: 0.20; 95% CI: 0.05-0.86) and those aged ≥65 years old (adj. HR: 0.24; 95% CI: 0.11-0.54), as well as those with low adherence to the Mediterranean Diet (adj. HR: 0.28; 95% CI: 0.14-0.56) or with hypertension at baseline (adj. HR: 0.34; 95% CI: 0.14-0.82). In addition, 10-year CVD attributable mortality was observed significantly less often in participants who consumed high, vs. low, intakes of small fish rich in n-3 fatty acids (4.3% vs. 1.1%; *p* = 0.015). Following potential confounding adjustment for several demographic, lifestyle and clinical variables, participants consuming >1 serving of small fatty fish per week exhibited a 76% decreased 10-year risk of dying due to CVD (adj. HR: 0.24; 95% CI: 0.06-0.99). As illustrated in Figure 2B, this beneficial impact was most prominent among those aged ≥65 years old (adj. HR: 0.22; 95% CI: 0.07-0.46) and women (adj. HR: 0.36; 95% CI: 0.05-0.62) and, as well as those with low adherence to the Mediterranean Diet (adj. HR: 0.22; 95% CI: 0.05-0.92) or without hypertension at baseline (adj. HR: 0.31; 95% CI: 0.13-0.73). When the analyses were focused on 20-year CVD mortality, similar but not significant associations were observed (all *p*-values >0.10).

## 4 Discussion

The present study is the first of its kind to evaluate the long-term effects of consuming seafood and/or small fish rich in n-3 fatty acids on mainly 10-year, and less prominently on 20-year, CVD incidence and mortality in a community dwelling Mediterranean population. The study findings revealed that less than one-third of participants adhered to the Greek National Dietary Guidelines recommendations and consumed >2 servings of marine fish and seafood on a weekly basis, while less than one-10th consumed >1 serving of small fish rich in n-3 fatty acids per week. Participants who consumed >2 servings per week of seafood had at least 27% decreased risk of developing CVD and 74% lower 10-year risk of dying due to CVD. Finally, participants with high consumption (>1 serving/week) of small fish rich in n-3 fatty acids had a significantly reduced risk of 10-year CVD incidence and exhibited a 76% decreased 10-year risk of dying due to CVD, even among normotensive individuals. When the analyses were focused on 20-year CVD incidence and mortality, similar but not significant associations were detected. The above findings highlight the importance for augmenting the consumption of seafood, and particularly small fish rich in n-3 fatty acids, for deterring particularly both 10-year CVD incidence and attributable mortality in the general population, including among those without apparent excess CVD risk (i.e., normotensive individuals).

Fish and seafood consumption varies greatly across Europe, with white fish representing 49% and 45% of the intake of total fish in women and men, respectively. Highest consumption levels and variability in types of fish consumed are detected in Mediterranean countries, including Greece. However, highest fatty fish intake is observed in Northern European and Scandinavian countries (Welch et al., 2002), despite the fact that in Mediterranean countries small fatty fish are abundantly found and relatively less expensive than their white fish counterparts, as well as regularly consumed within the context of the local traditional Mediterranean Diet. The study findings revealed limited adherence, corresponding to approximately 30% and 10%, to current Greek National Dietary Guidelines recommendations regarding seafood (>2 servings/week) and small fatty fish (>1 serving/week), respectively. While increasing trends in seafood consumption have been documented in the Greek population (Skourlis et al., 2020), adherence to the National Dietary Guidelines necessitates further encouraging the consumption of small fatty fish through related public health promotion strategies.

To date, dietary guidelines targeting CVD prevention often entail both overall and fish specific intake recommendations, albeit with varied target audiences. Specifically, the American Heart Association (AHA) encourages the consumption of 1–2 seafood based meals per week to reduce CVD risk and death (Rimm et al., 2018). Furthermore, the Sixth Joint Task Force of the European Society of Cardiology and Other Societies on Cardiovascular Disease Prevention in Clinical Practice recommend that in addition to consuming seafood 1 to 2 times per week, one such event should entail the consumption of fatty fish (Piepoli et al., 2016). Finally, based on the above, the AHA recently recommended omega-3 PUFA supplementation in patients at CVD risk, including those with CHD, type 2 diabetes mellitus (T2DM), and/or heart failure (Siscovick et al., 2017). To date, similar recommendations for the general population remain to be determined due to a paucity of conclusive evidence regarding marine n-3 fatty acid intake and long-term CVD outcomes in the general population (Siscovick et al., 2017). Contradictory findings question the utility of recommending high intakes of fish rich in n-3 fatty acids, as opposed to total seafood, for primary and secondary CVD prevention in healthy individuals (Giosue et al., 2022), and particularly in light of risk assessments regarding potential contaminants present in large fatty fish (Ferrante et al., 2019; Danopoulos et al., 2020). Moreover, meta-analytic findings fail to discern whether such recommendations could deter CVD in healthy individuals (Mohan et al., 2021), whereas juxtaposing evidence suggests that such recommendations should be limited to specific patient groups at augmented CVD risk, rather than the general population. For example, whilst on the one hand T2DM and vascular disease patients apparently benefit from even modest seafood consumption in deterring CVD risk (Jayedi et al., 2021) and mortality (Mohan et al., 2021), on the other hand such beneficial effects are not sustained in CHD patients (Steur et al., 2021), let alone healthy individuals. Finally, the above are further convoluted by meta-analyses of clinical trials showing that the efficacy of omega-3 fatty acid supplementation on CVD outcomes in healthy individuals varies from beneficial to negligible (Hu et al., 2019). Hence, further evidence regarding the long-term effects of high fish intake rich in n-3 fatty acids in deterring CVD and related adverse outcomes in healthy individuals is necessary (Siscovick et al., 2017) for informing the development of optimal dietary guidelines for cardiovascular health (Kwok et al., 2019) in the general population.

Regarding the impact of seafood and fatty fish intake on deterring 10-year incidence of CVD, the present study findings showed that participants who consumed >2 servings per week of seafood had the most prominent beneficial effects, exhibiting at least 27% decreased risk of developing 10-year CVD. These findings were upheld also in relation to small fatty fish intake most predominantly among at risk subpopulation groups, such as the elderly, those with low adherence to the Mediterranean Diet, and/or with hypertension. To the best of our knowledge, similar results regarding 10-year CVD have not been documented in the literature to date. Even so, our findings corroborate with similar results from recent umbrella reviews showing that increasing fish consumption reduces the risk by 12%–25% of an array of incident CVD events, including heart failure (SRR: 0.80; 95% CI: 0.67-0.95), myocardial infarction (SRR: 0.75; 95% CI: 0.65-0.93), and CHD (SRR: 0.88; 95% CI: 0.79-0.99) (Jayedi and Shab-Bidar, 2020). Furthermore, they partly agree with previous findings showing that high intakes of fish rich in n-3 fatty acids, as opposed to total seafood, may not in fact deter CVD incidence (Giosue et al., 2022) particularly in healthy individuals (Mohan et al., 2021) since while small fatty fish consumption did not significantly reduce 10-year CVD events in the overall population, it notably reduced such risk in subpopulation groups such as the elderly, those with unhealthy diet quality, and/or with hypertension.

The decreased CVD mortality risk that was observed in participants consuming >2 servings of marine fish and seafood on a weekly basis was notable in subpopulation groups at increased CVD risk, such as women, the elderly, and those with low adherence to the Mediterranean Diet. However, it is noteworthy that high intake of small fish rich in n-3 fatty acids was associated with a reduction in CVD mortality even in otherwise healthy normotensive individuals. Previous reports show that high intakes of fish rich in n-3 fatty acids may decrease the risk of CVD mortality (Jayedi and Shab-Bidar, 2020) in healthy individuals (Mohan et al., 2021), albeit approximating solely a 4% reduction in risk (RR: 0.96; 95% CI: 0.94-0.98) (Kwok et al., 2019). A dose-response relationship in this association is revealed by recent umbrella reviews supporting that for every 100 g/d increase in fish consumption a corresponding reduction by 25% in CVD mortality risk follows (Jayedi and Shab-Bidar, 2020). To the best of our knowledge similar findings regarding n-3 fatty acid fish consumption and 10-year CVD mortality have not been reported to date.

Regarding the subpopulation of older participants aged >65 years, it should be mentioned that during senescence, among other characteristics, lifestyle habits are modified (Groessl et al., 2019; Eckstrom et al., 2020; Tsou et al., 2022). A reduction on the leisure-activities, mainly due to mobility problems, is observed, with most older adults not meeting the currently recommended minutes of regular physical activity per week (Groessl et al., 2019; Eckstrom et al., 2020). Moreover, dietary habits become less diverse; mainly due to dysphagia and swallowing problems or economic reasons, a reduction in the consumption of some foods, such as fruits and vegetables, fish or seafood, is frequent (Steenhuis et al., 2009; Tsou et al., 2022). Thus, based on our findings, increasing the consumption of seafood and small fish might be particularly beneficial for this age group.

The beneficial health impacts of consuming small fish rich in n-3 fatty acids on CVD health are primarily attributed to their rich EPA and DHA content, as well as their elevated composition of minerals and vitamins known to enhance cardiometabolic health, such as calcium, phosphorus, magnesium, zinc, and vitamin D (Petsini et al., 2019). High n-3 fatty acid intake has been associated with favorable cardiometabolic profiles secondary to enhanced triglyceride lowering effects, endothelial function, and antithrombotic effects, as well as lower oxidative stress and inflammation (Gajos, 2019; Panagiotakos and Kouvari, 2021). Specifically, n-3 fatty acid intake apparently mitigates adverse cascades entailed in the underlying biological pathways of lipoprotein levels, blood coagulation, and/or blood pressure levels, which in turn collectively ensure the maintenance of CVD health (Kris-Etherton et al., 2002; Panagiotakos et al., 2005). Since previous studies have shown that regular fish consumption is associated with decreased synthesis of proinflammatory markers levels (such as IL-6, CRP and TNF-alpha) (Zampelas et al., 2005) which are pivotal elements of the aforementioned biological pathways, it is upheld that high fish intake rich in n-3 fatty acids may diminish an inflammatory immune response state which would otherwise favor CVD onset and/or further progression. A “U-shaped” dose response relationship is likely entailed, with lowest cytokine production being recorded following 1.0 g/day n-3 fatty acid supplementation (Trebble et al., 2003). While initial reports suggested that the optimal intake of n-3 fatty acids for maximum reduction in entailed inflammatory processes was 0.6 g/d, the present study findings suggest that in community dwelling individuals at least one serving per week (approximating 1.5–2 g of n-3 fatty acids (Mozaffarian and Rimm, 2006)) is associated with a significant reduction in adverse CVD outcomes.

In summary, the cumulative results arising from observational studies regarding fish intake, randomized clinical trials regarding fish oil supplementation, and related experimental studies suggest that regular fish consumption may decrease the incidence of coronary heart disease or acute coronary syndrome, albeit with uncertain impacts on total CVD events (Wang et al., 2006; Brunner and Iso, 2008; Siscovick et al., 2017; Jayedi and Shab-Bidar, 2020; Panagiotakos and Kouvari, 2021) in the general population. In particular, a pooled analysis of four large-scale international cohort studies, including 191,558 people from 58 countries, indicated that minimal fish intake (approximately 2 servings or 175 g weekly) is associated with lower risk of major CVD events and attributable mortality among patients with prior CVD, but not in the general population (Mohan et al., 2021). Moreover, clinical trials regarding n-3 supplementation in CVD-free individuals, such as the VITAL-placebo controlled trial where the intervention group was provided with 1 g/day marine n-3 PUFAs, after a median follow-up of 5.3 years, no beneficial effects on major CVD events were observed, except in individuals with low reported fish consumption at baseline (Gajos, 2019). In contrast, our results suggest that adequate intake of fish rich in n-3 fatty acids may induce 76% decreased risk in 10-year CVD mortality in the general population, and respectively 69% diminished risk even among low risk normotensive individuals. Therefore, public health promotion interventions aimed at deterring CVD ought to promote the intake of seafood, and particularly fish rich in n-3 fatty acids, in the general population, including normotensive individuals at low risk for adverse CVD outcomes, *via* related targeted public health interventions.

### 4.1 Strengths and limitations

The study strengths include the prospective cohort study design adopted and the representative randomly selected, population-based sample selected living in the most densely populated district in Greece where CVD prevalence rates are notably elevated (Panagiotakos et al., 2007; Aunan et al., 2016). In addition, the lengthy duration of follow-up deters a misclassification bias being introduced due to disease latency and hence allows for the accurate assessment of CVD related outcomes. Furthermore, the dietary assessment method employed allows for the accurate evaluation of overall fish consumption which overrides seasonal and/or other temporal variations. However, the study limitations include that dietary assessment was limited to a single baseline measurement. Hence, changes in dietary patterns over time and/or complete dietary analysis for nutrient components was not assessed. Despite this fact, it is posited that overall dietary patterns, such as that assessed by the MedDietScore, are not likely to significantly change over time. Even so, fish intake was evaluated by self-reported FFQ and hence information regarding the amount of fish consumed could be over- or underestimated. Another limitation is the small number of individuals who consumed very high levels (>300 g/week) of fish. Additionally, when the analyses were focused on 20-year CVD incidence and mortality, similar but not significant associations were detected, albeit likely due to limited study power mandating further investigations in large scale populations with extended follow-up periods. Finally, the present analysis did not account for pharmaceutical treatments and their mediating effects upon CVD outcomes. However, it is upheld that any such treatment effects would only bias the present results towards the null hypothesis. Therefore, it is anticipated that the present findings are an underestimation of true effect sizes.

## 5 Conclusion

The present study revealed that participants who regularly consumed seafood exhibited a decreased risk of developing and/or dying from CVD. Therefore, public health promotion interventions aimed at deterring CVD ought to promote the intake of seafood, and particularly fish rich in n-3 fatty acids, in the general population, including normotensive individuals at low risk for adverse CVD outcomes, *via* related targeted public health interventions. Further clinical trials are necessary for elucidating the efficacy and appropriate fish intake levels for securing optimal CVD health particularly in otherwise low risk normotensive individuals.

## Data Availability

The raw data supporting the conclusions of this article will be made available by the authors upon request.
